# Inside regular lab meetings: The social construction of a research team and ideas in optical physics

**DOI:** 10.1177/03063127231188132

**Published:** 2023-08-22

**Authors:** Axel Philipps, Laura Paruschke

**Affiliations:** Leibniz Universität Hannover, Germany

**Keywords:** lab meeting, research team, forms of presentation, research implicature, participant observation, optical physics

## Abstract

Scheduled meetings are associated with standardization and understood as a bureaucratic form of coordination, control, and rule observation. In attending assemblies of a research team in optical physics for over a year, we found regular lab meetings are compulsory for all their members and are an avenue to announce and give information about new and changed institutional regulations, to supervise members’ activities and their output. But more importantly, they offer an environment for continuous thinking through talk that goes beyond announcements. Meetings are a protected space to comment on conducted research, to amend experimental set-ups, to test argumentation, and to outline potentially new directions of research. By participating in these practices, researchers, become members of the team as they get acquainted with the ongoing research; its scope, problems, and limits; the solutions at hand; and the know-how within the team. In functional terms, observed internal meetings seem to (a) ensure that the research team focuses on a specific research agenda by talking about and discussing ongoing research in the lab, (b) be used to discuss and assure the quality of the team’s research output, and (c) generate and inspire new research within the team. Our findings suggest regular internal meetings, like shop talk, are constitutive of doing science by talking about ongoing research.

Working in research teams is standard practice in most branches of science today (Leahey, 2016; Wuchty et al., 2007). Teams are essential for carrying out complex and laborious study designs. Research on research teams mostly concentrates on the team’s size and its organization and how it impacts outcomes, careers, and innovativeness (Carayol & Matt, 2004; Cook et al., 2015; Heinze et al., 2009; Horta & Lacy, 2011; Lee et al., 2015; Mote et al., 2016; Perović et al., 2016; Qurashi, 1991; Rey-Rocha et al., 2006; Seglen & Aksnes, 2000; Wu et al., 2019). These studies usually take for granted that research teams are creative units that work and publish together. But how does a research team become a productive and creative unit? How do researchers build a team that is more than individual scientists who sometimes talk and publish together? In following a research team in optical physics for over a year, we found regular lab meetings to be critical in socially constructing a research team and producing research ideas.

In existing accounts, meetings are typically understood as a bureaucratic form of coordination, control, and rule observation, and are associated with standardization (van Vree, 2011; Walsh & Lee, 2015; Weber, 1978). In line with this literature, the meetings of the research team we observed are compulsory for all its members, and are an avenue to announce and give information about new and changed institutional regulations and to control members’ activities, output, and progress. But, more importantly, the ordered talk in the meetings, we argue, also provides an environment for thinking through scientific matters. Meetings are a protected space to amend experimental set-ups, to test lines of argumentation, and to outline potentially new directions for research. Through being part of these practices, researchers become members of the team because they become acquainted with the ongoing research, its scope, problems, limits, potential solutions and the know-how within the research team. Moreover, the regular meetings consolidate the team and their research as a whole. In a nutshell, the meetings we observed function to:

(a) ensure that the research team focuses on a specific research agenda by talking and discussing ongoing research in the lab,
(b) discuss and assure the quality of the team’s research output, and
(c) generate and inspire new research within the team.

We argue that all these aspects offer an environment of tacit control, moderation, and inspiration that is more effective than strong leadership (Heinze et al., 2009) alone or guidance ‘by the iron hand of the scientist who organized laboratory work’ (Latour & Woolgar, 1986, p. 127).

In this paper, we develop our understanding of regular meetings as being fundamental to doing science and forming research teams. Instead of researching organizational contexts and the characteristics of research team members, we closely examine presentations made in meetings and how participants discussed about ongoing research in meeting talk. We argue that just as shop talk (Lynch, 1985) is constitutive of doing research, so are regular internal meetings. Our conclusions build on a detailed and comprehensive description of structural, procedural, and discursive characteristics of observed meetings of a research team in optical physics.

## Research on research teams and laboratory shop talk

Research teams are a recurrent subject of organizational studies. Scholars in this field have especially examined the causal effects of organizational factors on productivity and creativity (Barjak & Robinson, 2008; Carayol & Matt, 2004; Cook et al., 2015; Fiore, 2008; Heinze et al., 2009; Horta & Lacy, 2011; Kretschmer, 1985; Lee et al., 2015; Mote et al., 2016; Perović et al., 2016; Qurashi, 1991; Seglen & Aksnes, 2000; Wu et al., 2019). These studies have shown that outcomes depend on the size and composition of research teams as well as on strong team leaders who provide compelling goals, keep researchers focused and motivated, and offer opportunities to enhance team members’ capacities of active listening, being open minded and able to communicate clearly. They concentrate on the effects of independent variables (e.g., size, degree of diversity) and the demands on leadership, but tend not to address the processes of how scientists jointly conduct research and produce scientific outcomes.

Laboratory studies, in contrast, have closely observed and analysed research practices (e.g., Knorr Cetina, 1981; Latour, 1987; Latour & Woolgar, 1986; Lynch, 1985). These investigations replaced the idea that nature speaks for itself by introducing the concept of socially constructed scientific facts. In this view, facts are readable signs produced and measured with instruments scientists envision and build. Consequently, each instrument only represents the current state of methods and theories. Moreover, laboratory studies have found that scientists put as much effort into the production of scientific facts as they do on producing texts. They have shown that scientists devote considerable energy applying stylistic, grammatical, and lexical features in scientific reports to transform in some way ‘messy’ and contingent practices into impersonal, procedural routines in laboratory work (Gilbert, 1976; Gilbert & Mulkay, 1984; Knorr Cetina, 1981; Latour & Woolgar, 1986; Lynch, 1985). In fact, since the early days of science, researchers have introduced various linguistic resources and rhetorical strategies to persuade fellow scientists and other readers (Bazerman, 1988; Gross et al., 2002; Myers, 1989). Science produced its own distinct text genre with scientific articles (Swales, 1990), and peer review became the standard to control the way in which scientists present ideas and findings (Chubin & Hackett, 1990).

While scientific publications communicate knowledge and authors are acknowledged for their contributions, scientific communication includes much more than published texts. For example, ethnomethodologist Lynch (1985) highlighted shop talk as an integral part of the ongoing laboratory work of making scientific knowledge. Structural features of talking science hardly differ from ordinary conversations. However, laboratory shop talk accompanies the ongoing production of a scientific inquiry. Through shop talk, researchers align their interpretation of what is going on in the lab and make sense of a record’s analyzable features by identifying artifacts in their lab work. Elsewhere, Lynch (1982) spoke of *critical inquiries* as instances of laboratory shop talk. For example, he examined a conversation between two scientists about a produced micrographic montage and whether it was useful for the ongoing project. He contrasted such shop talk—an integral part of situated lab work—with scientific reports, ‘seminars or conferences where colleagues formulate and reformulate each other’s accounts of their work and its finding’ (Lynch, 1982, p. 512). Instead of strategically debunking the arguments of rival researchers, he argues that shop talk in lab research is more ‘tied to the “object” under investigation’ and that ‘scientists are preoccupied with getting the day’s work done’ (Lynch, 1982, p. 512).

Like Lynch, Amann and Knorr Cetina (1988, 1989) studied situated conversations between scientists about how to interpret the data from an autoradiograph film. In their investigation of spontaneous talk during laboratory work, they found language devices that also appear in ordinary conversations to organize interactions and verbal exchanges. These include structural features of conversations, such as opening sequences (e.g., greeting and acknowledgement), inquiry sequences (e.g., question-answer, assertation-confirmation), evaluation and closing sequences of inquiries (e.g., performance recommendation). They described these devices as ‘inference-producing’ in that they arrive ‘at (temporarily) definitive readings of the sense data their equipment turns out’ (p. 11). They further differentiated between *oppositive exchange* and *procedural implicature*. In an oppositive exchange, speakers highlight problems in an ongoing discussion or make apparent features of a phenomenon not previously recognized. Applying adversarial devices, participants ‘argue with, expand upon, and negotiate candidates’ accounts and formulations’ (Amann & Knorr Cetina, 1989, p. 15). In conversational implicatures, what a speaker says literally is distinct from what is meant—one has to include context to infer meaning in a conversation. A feature of procedural implicatures that is especially relevant for research contexts is that the conclusions generated through inference ‘need not provide an answer to the opening sequence which may have been a problem statement’ (Amann & Knorr Cetina, 1989, p. 13). Rather than going through the procedural history of particular phenomena, speakers produce nonobvious conclusions in the form of interpretations or performative recommendations.

In all of the aforementioned investigations of scientists’ conversations, the focus has been on situated talk in the course of laboratory work. Social scientists of science have mostly studied instances of shop talk that were embedded in casual conversations taking place at benches, in offices and hallways. Various scholars have mentioned regular meetings of scientists to talk about current research (Knorr Cetina, 1981; Latour & Woolgar, 1986; Lynch, 1985; Stephens & Lewis, 2017), but they have not studied this form of ordered talk in depth. When Stephens and Lewis (2017) called for more research on ordered talk in scientific meetings, they stated that meetings are key sites to study interaction in science, such as the performative displays of disciplinary identity in an interdisciplinary setting.

In the broader sociological and research policy literature, regular meetings have been treated as devices of control and standardization—for example to coordinate inquiries—but have seldom been seen as part of doing research through talking (van Vree, 2011; Walsh & Lee, 2015; Weber, 1978). In this paper we argue that ordered talk in regular internal meetings is, like shop talk, constitutive of doing science and, moreover, of forming a research team. Such teams often are clustered in different laboratories, topics, and expertise because laboratory work is mostly organized in groups with different specialties and concerns. The making of a research team begins under the umbrella of a research agenda. But more important is that in the clustered settings, scientists take part in casual conversations related to the local work scene. Scheduled gatherings might stop scientists from doing experimental work and engaging in ad hoc conversation, but meetings provide space to reflect on ongoing research in the team. In regular meetings, team members from different corners and fields of the lab come together, get informed and exchange their current research, which shapes the team and its future research.

## Research background

To elaborate our argument, we will take a closer look at how participants’ talk constructs and consolidates their team and research during regular internal meetings. We will focus on a research team’s hour-long meetings held one morning a week, over video conference or in person. The team we observed is one of six in a university’s department of optical physics; all six teams are doing research on the interaction of light with different matter. The specific research agenda of the observed research team is closely associated with the head of the team. When appointed as full professor, he established the team and equipped the laboratories, which are now organized into different groups, doing fundamental and applied research with a particular light source. One group studies characteristics of this source theoretically, and the others do so experimentally in two laboratories on different floors. Each lab consists of various moveable apparatuses and tables fixed to the ground, which host different experimental set-ups. While most of the bachelor, master, and doctoral students can be found around these tables, senior scientists, including the head of the research team, a full professor, usually work in their offices. All members of the research team work in the same wing of the university building, which allows them to communicate spontaneously if needed.

**Figure 1.**
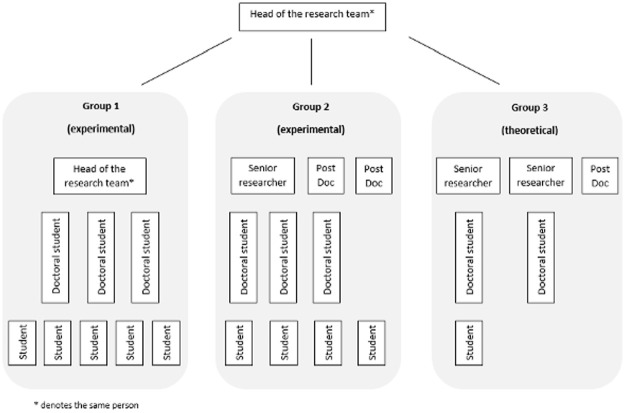
Schematic overview of the observed research team.

During our stay with the research team, there were around 25 team members, including students. The size of the research team changed: some students left and others (including a postdoc) joined the team during our observation. The research team comprised a full professor, senior staff scientists, postdocs, doctoral, master’s and bachelor’s students. Apart from members’ academic status, the research team can be differentiated into three groups, which are hierarchically organized within the team (see Figure 1). There is the research team leader, who presides over one theoretical and two experimental units. During our time, the leader supervised his own group of students doing experimental physics whereas the senior scientists were responsible for the two other units. The theoretical sub-group included two senior scientists, a postdoc, doctoral and undergraduate students. The experimental physicists constituted another subgroup with a senior scientist, two postdocs, doctoral students, and most of the BA and MA students. Some members of the research team are also involved in other research clusters at the university and beyond. Regarding employment status, the full professor and one senior scientist had permanent positions, whereas all others had fixed contracts. Five out of 25 researchers were women (mostly degree candidates, doctoral students, and one postdoc); this is typical for departments of physics at German universities, which were 19 percent women in 2019 (DFG, 2021). Due to foreign team members, the common language in and outside of meetings was English.

## Data and methods

One of the authors attended weekly meetings of the research team for more than a year, and the other visited six meetings on campus. In total, we joined 45 online and in-person meetings in full length. In this paper, we focus on the observations of 39 online gatherings between October 2020 and October 2021.

At the beginning of our fieldwork, we planned to conduct participant observations in labs and meetings and to do qualitative interviews with members of the research team. When we started our fieldwork in mid-October 2020, we had only a few opportunities to visit laboratories before access was restricted due to the pandemic situation and lockdowns that began from December 2020 onwards. The observed scientists could visit their offices but were advised to work at home whenever possible. To continue experiments, only two researchers wearing masks were allowed in the laboratories.

These restrictions were one reason why our attention shifted towards the research teams’ internal meetings. Another motivation was an interview with the head of the team, who spoke about opportunities to talk about ongoing research before and during the lockdown. He reported that they often discuss problems and ideas around research ad hoc in the lab, over lunch and in meetings:

*HD*^1^: It’s just that you meet in the lab and talk about different things. My communication used to take place mainly on the way to the canteen. So, before the pandemic, we went to the cafeteria as a group, ten people or so. […] Then, I always choose one and then talked to them more intensively about it [their research]. And that’s just very important, such conversation and to see what are the current worries and problems that arise there [in their research]. Yes, or you can organize meetings. Everyone comes together, everyone tells what he is doing, where he is currently attached to, where the problems are and then the others should also comment on it and maybe a good new idea arises, or things can be solved very easily.

### We concentrated our further observations mainly on the online meetings

After an introduction to all team members, and with their consent, we attended the meetings until gatherings took place in person again. During the online meetings, one of the authors participated muted and with the video camera turned off. He joined the meetings a few minutes before the official start and stayed until the end when all members had left. In addition, we joined meetings in face-to-face settings in 2022 to further understand the specificity of attended online meetings. It became apparent that interaction on campus differed slightly. For example, participants were present during online meetings in form of icons as well as visually and audibly if they presented a topic or took part in a discussion, but in the campus room they attended the meetings physically and gathered in small groups after the meetings had formally ended. In these groups, some continued their exchange with the presenter in more detail; others used the opportunity to talk with colleagues about different topics (e.g., administration, teaching). Such post-meeting talk was not common in online gatherings. In two cases, a small number of online meeting attendees stayed for prolonged discussions; however, these additional exchanges were planned in advance of the online meetings. They did not occur spontaneously, and if some team members met after an online meeting, it was not accessible to us. However, in some cases, we know of research groups which met after the official meetings in privately organized online rooms. Very few pre-meeting conversations (Raclaw & Ford, 2015; Yoerger et al., 2015) took place in the campus room or in online meetings. Most participants appeared only a few minutes before, or right on time for, the meeting. On campus, they went straight to a free seat, and if they arrived in a group, they mostly stopped their small talk. However, this small talk indicates that attendees met before in their offices and the hallways. In the online space, they joined muted with the video off. Only a few—usually the professors—entered and said hello to everyone, but they also turned off their video and microphone afterwards.

In general, before the official start of (in-person and online) meetings, the only visibly active people were the presenter and one other person checking technical issues. However, after these tests, the latter stepped down or vanished, muted behind dark screens. On time at 9 o’clock on campus the moderator—the head of the research team or a senior scientist—spoke up, and in the online format he turned on his video and started the meeting with an official welcome. In the online setting, he stayed visible for the entire meeting, whereas others only turned on their videos after the presentation if they asked questions or when they took part in a discussion. Apart from these differences between online and face-to-face meetings, the greatest differences were post-meeting conversations when, instead of dark screens, attendees physically joined dynamic and enthusiastic small discussion groups in the room. Such conversations offered further occasions to continue and deepen the exchanges about ongoing research, for example, through explicit follow-up questions that were not suitable for the previous discussion.

During our observation, we took notes, recorded discussions, and consulted members of the research team if we had questions about the meetings. We wrote down who presented on what topic, how the meeting evolved, who said what, who and how participants took part in discussions, and finally the number of attendees. In addition, we registered changes in the structure and order of meetings, which included timestamps. Discussions with many turns and oppositive exchanges were transcribed and closely analyzed. Finally, we read and compared published papers of the research team with presentations given during our stay with the research team to better understand features of meeting presentations. The arguments presented here are part of a research project on researchers’ ways of dealing with unforeseen events during experiments. Its focus is on laboratory research in physics and biochemistry. In this broader project framework, social researchers joined research teams in their labs and conducted problem-centered interviews with them about their educational and research background, their experiences with and handling of unforeseen events during their experiments and their understanding of success and failure (see Barlösius & Philipps, 2022). In this paper, we concentrate on the observations of the regular online meetings to show what appeared in our data and to classify such meetings in research contexts. These characteristics will be presented in detail by looking at different aspects of the meetings, ranging from analyses of all meetings to reviews of particular sequences of talks.

## Composition of meetings

Attending members of the research team joined online meetings from their homes, and only a few attended from their offices. On some occasions, visitors from other research teams and universities took part in the meetings. These guests were usually partners from other universities with whom scientists of the research team cooperated. Thus, one could say the regular gatherings were mostly internal meetings. In the 39 meetings observed, 23 members attended on average (though in-person meetings averaged closer to 15). Most of the time all postdocs and senior scientists appeared, and variations from one meeting to another were due to the changing numbers of undergraduates (from four to ten) and doctoral students (from five to 12). All members of the research team who had contracts with the university attended on a regular basis.

Team members participated differently in the regular lab meetings depending on their role. Most of the time, undergraduate and doctoral students reported on their research and only a few professors and postdocs contributed with presentations. During our observation, over the course of different meetings, 11 degree candidates and 11 doctoral students gave 30 presentations between them, mainly about their ongoing research and findings. In contrast five postdocs and four senior scientists gave between them nine presentations about the current state of research and concepts. The proportion of contributions reversed, however, in discussions. Senior scientists especially provided comments, asked questions, and exchanged information about the subject. In 35 meetings with scientific presentations, for example, senior scientists took part in all discussions, whereas postdocs only contributed with comments to 11 meetings. Doctoral students provided insights from their experiments or asked questions in 16 meetings and undergraduate students in two meetings. Counting the number of contributions of the participants in the subsequent discussions, a similar pattern emerged; senior scientists contributed the most, followed by doctoral students and postdocs. Similar proportions of participation of the different status groups occurred in the face-to-face meetings. This pattern of input and exchange coincides with observations made by Shinn (1982) who found that professors were responsible for conceptual work and overseeing experiments while doctoral students set up the experiments and executed them. However, both groups were continuously in exchange about the experimental progress. This division of labor was reproduced in the observed meetings. Doctoral students conducted and reported on their ongoing experiments, while postdocs and senior scientists gave insights into the state of research in their field of specialization and commented in general on presentations.

### Length of the meetings and topics discussed

The composition of and proportional contributions to the meeting did not affect the duration of a meeting. Each meeting was approximately one hour (on average 56 minutes). Times ranged from rather short meetings of 25 minutes to long of up to 93 minutes. In two cases a selected group of members stayed after the official meeting to further discuss group-specific subjects. However, meetings in general were used to present and discuss scientific subjects. On average 69 percent of the meeting time was dedicated to scientific presentations about recent developments in the research field or researchers’ experimental and theoretical work and 23 percent of the meeting time was spent in discussions related to these presentations. Only eight percent of the meeting time was used to make announcements unrelated to the scientific content. This meant that in most meetings only a few minutes were reserved for announcements. Nonetheless, this average time for announcements includes five meetings with extended period informing participants about current non-scientific developments such as changes in email and IT safety regulations, handling of virtual conference tools and reference management systems, and commentary from foreign team members on special events such as the celebration of New Year, which is celebrated differently in Asia.

The time and topic management of meetings already provides some insights into the observed regular lab meetings’ order and characteristics. Meetings are clearly research driven, with the main part being the scientific presentations and subsequent discussions; announcements play a minor part. Viewing meetings as a process offers additional insights into how the meetings’ organization helps to constitute the team and research ideas.

### The opening

Each meeting officially started when a moderator—usually one of the professors—turned on his video and microphone and said:

*HD*: So, now it is 9 o’clock. Good morning, everybody, and a warm welcome to our group seminar. Before we get started, are there any announcements? (Meeting 6)

When the moderator mentioned the time at the beginning of each meeting, he underlined its formal character. Regular meetings have a fixed-time schedule. In the case of the internal lab meeting, its formal character is also evident, as it is listed in the university’s lecture catalogue.

One should note that teaching duties are relatively high at German universities. In the observed research team, the full professor, for example, has to deliver five courses each term. Organizing lab meetings as seminars reduces such burdens and provides an opportunity to open their subject to students—in this case to students who write their bachelor’s and master’s thesis supervised by members of the research team. However, the observed internal meetings were unlike teaching seminars because most attendees were already members of the research team, and unlike research seminars because all presenters were lab members, rather than visiting speakers from other universities and research teams.

Regarding the meeting’s purpose, the regular lab meetings often went straight to their subject after the opening:

*HD*: Then let’s get started into science. Today, we have a talk by … (Meeting 6)

*HD*: Coming now to the scientific part … (Meeting 5)

A meeting’s organization usually left no space for personal communication or other topics. Moreover, the opening reveals that the meetings actually ‘get started’ after the announcements, which again indicates that, from the members’ point of view, their main purpose is not to inform about new regulations but to talk about science. Such meetings thus are far from being just a bureaucratic form of coordination and control.

### Presentations

It therefore is no surprise that 35 out of the 39 meetings observed were mainly reserved for scientific presentations and discussions. The undergraduate and doctoral students gave 27 presentations about their experimental work and findings, whereas the postdocs and senior scientists spoke in eight presentations mainly about concepts and theories. Four meetings were used for slightly different purposes. Three were entirely dedicated to test and amend presentations for scientific conferences. In these cases, lab members gave their conference presentations in advance and received feedback on their slides and verbal presentations. It was characteristic of these meetings that participants did not discuss the content of the presentation. Their comments were not about science but the presentation’s comprehensiveness, readability, and visual appearance. In one meeting, the head of the research team presented the concept of a new research building and all its facilities. The presentation was about the building’s architecture and function and not about current research and scientific issues.

A closer look at the scientific presentations shows that most of them followed structures that are typically used in presentations at conferences. All started with a title and contextual information (such as who contributed to the presentation) as well as the logo of the institution and of the funding organization (if applicable). Next, experimental physicists mainly provided overviews about current developments in certain research fields, technical changes and ongoing but early-stage research in the labs. Other speakers introduced new concepts and informed about new developments, challenges, and opportunities.

We observed three specific talk structures (see Table 1) that corresponded mainly to the presenter’s affiliation with either the theoretical or the experimental unit of the research team. Presentations about experiments, for example, started with a slide about the researchers’ motivation. Presenters offered information about the current state of research, gaps in that research, and theoretically and methodologically assumed possible outcomes. A detailed description of the experimental setup followed and was then supplemented with a presentation of findings, sometimes including reports on extended measurements and modifications to explain results. The presentation ended with concluding remarks and an outlook for further research. Theoretical presentations also began with overviews about the state of the art and open questions but, in contrast to empirical approaches, they continued with a conceptual idea and how it could be represented in equations and implemented via specific algorithms. Often such presentations also gave information about certain parameter tests and preliminary outcomes of simulations. The presentations finished with a conclusion on how to possibly translate concepts and ideas into experimental setups. Researchers chose a third form of presentation if they were reporting about early-stage experiments or giving an overview. After presenting the current state of (their) research, the presentation concentrated on preliminary findings, prior research, and associated quandaries and challenges. This type of presentation aimed to present problems and invite the research team to search together for solutions and promising research trajectories.

**Table 1.** Overview of three different structures of presentation.

Experimental	Theoretical	Report/Overview
Motivation	Motivation	Current state of research
Experimental setup	Conceptual idea	Challenges
Findings	Implementation	
Conclusion	Conclusion	

Most presentations were, in form and content, akin to presentations and published papers for an audience outside the research group. For instance, all data were already prepared and processed as facts in the form of numbers, diagrams, and graphs. Furthermore, presenters related concepts and findings to certain fields of research, highlighting gaps in the literature, the goals of their research, and the potential impact of their findings, much as they would in a published paper or conference presentation. However, presentations were not identical to these polished outputs: all presenters gave more information about their conceptual work, experiments, and findings, and spoke about practical insights during the meetings, much more than one can read in the researchers’ published papers.

Based on the above, we argue that regular lab meetings show three characteristics. First, a central feature of such meetings in research contexts is that participants talk scientifically. Scientists can probe disputed subjects within their research team, and young scientists in particular can familiarize themselves with the way in which arguments are arranged and findings are presented in their research field, by doing research and presenting it to their research fellows.

Second, concepts and findings are offered in a way that team members can comment and deliberate on them as friendly peers. In this respect, regular lab meetings are institutionalized forms of ‘para-collaboration’, coordinating researchers ‘working *alongside* one another’ (Hackett, 2005, p. 792) in contrast to collaborating in different experiments towards the same end. The meetings enable para-collaboration by providing a protected space with empathetic colleagues to talk about research and to exchange knowledge, as well as to keep it in line with the research agenda of the team. Moreover, they function as a way to control the output of the research team before it is exposed to the wider scientific community where it might harm the reputation of the research team if the findings are false or underdeveloped.

Third, all members of the research team get exclusive access to recently produced concepts and findings in the groups. They can become familiar early on with such research outcomes and use them in their own research. They also offer opportunities to develop presented concepts and findings further in a direction beneficial to the entire research team. The second and third characteristics fully unfold in combination with the subsequent discussions at the end of each presentation; the follow-up exchanges in particular prepare the ground to spread and develop ideas within the research team whilst strengthening the team’s research agenda.

### Subsequent discussions

In general, the observed verbal exchanges in the regular lab meetings reveal structural elements familiar from shop talk described in Lynch (1985) and with repertoires of ‘*production devices* for generating knowledge’ (Amann & Knorr Cetina, 1989, p. 11, emphasis in original). There are inquiry sequences (e.g., question-answer) as well as oppositive exchanges about experimental setups and produced results. Inquiries are often concerned with questions about unclear aspects of applied procedures (e.g., ‘Did cloaking happen?’, Meeting 10; ‘What steps are necessary to clean up the signal?’, Meeting 13; ‘I don’t understand why do you use this transformation step?’, Meeting 28). In further cases, research team members in the audience take presented ideas or results and apply them to other contexts and in different conditions. With questions such as ‘How sensitive is your network to dispersion parameter?’ (Meeting 12), ‘Is it possible to obtain higher order of …?’ (Meeting 33) and ‘What are bad materials? What are good detectors?’ (Meeting 35), they inquire into the potential value of the presentation for their own research, which goes beyond inquiries to understand and interpret data.

These inquiry sequences and oppositive exchanges produced *implicatures* for how team members understood the data and directed attention to previously unseen features of a phenomenon. There were also sequences in which participants’ discussion made implicatures for *new* research directions. As Amann and Knorr Cetina (1988, 1989) have previously observed, conversations contained recommendations to improve measurements and visualizations—what they termed procedural implicatures. However, in subsequent discussions we also identified spontaneous suggestions to develop ideas and prepare further experiments. These included newly created conceptual relationships that opened up new perspectives on the research but did not necessarily include instructions concerning how to translate a new perspective into experimental set-ups. These research implicatures in the form of a recommendation emerged from argumentative (oppositive) exchanges about the potential of the presented findings for further research. The sequence from Meeting 39 offers examples of procedural and research implicatures:


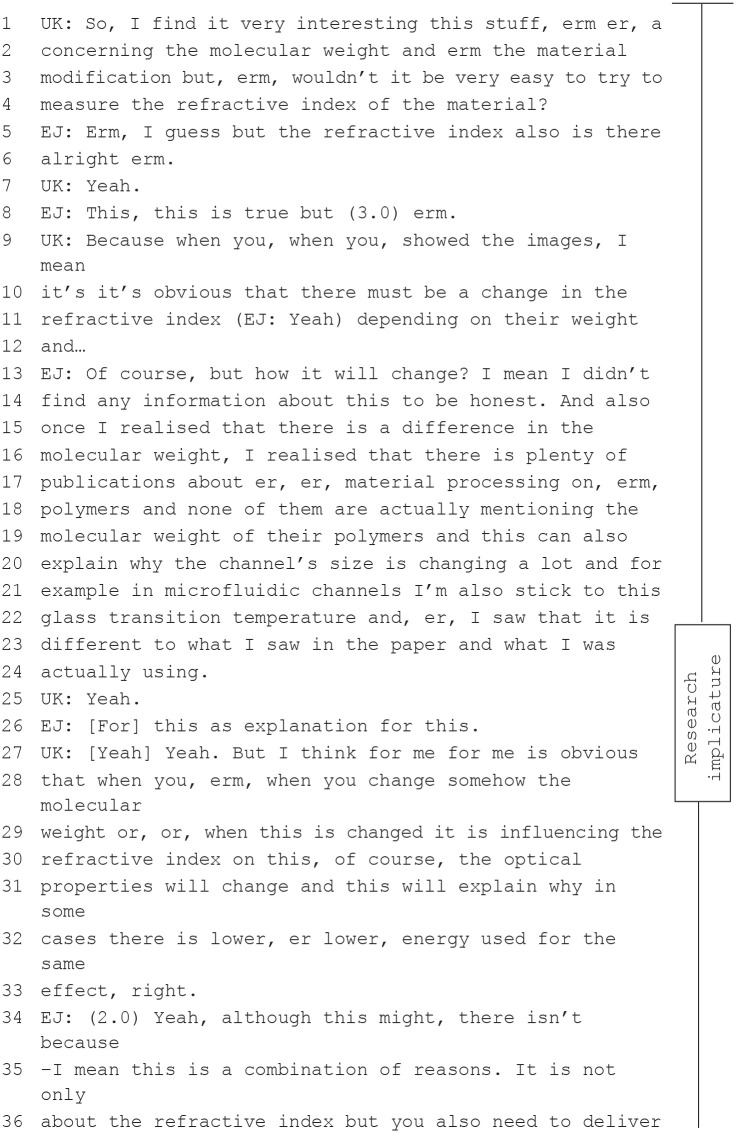



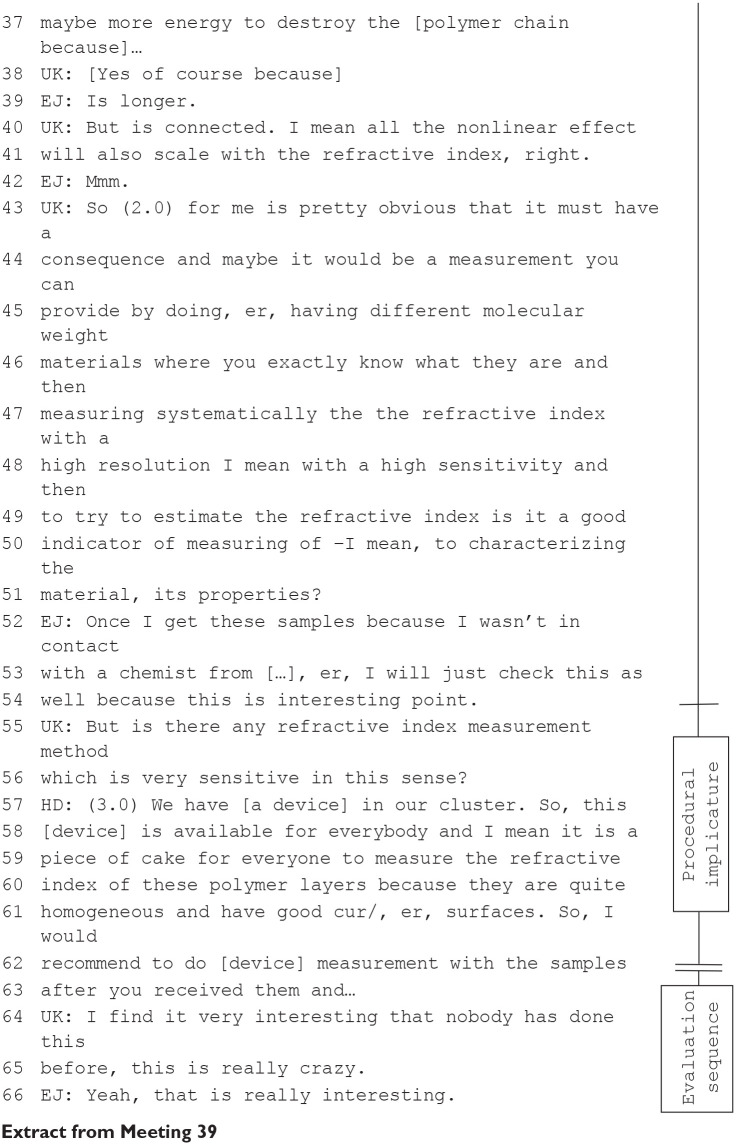



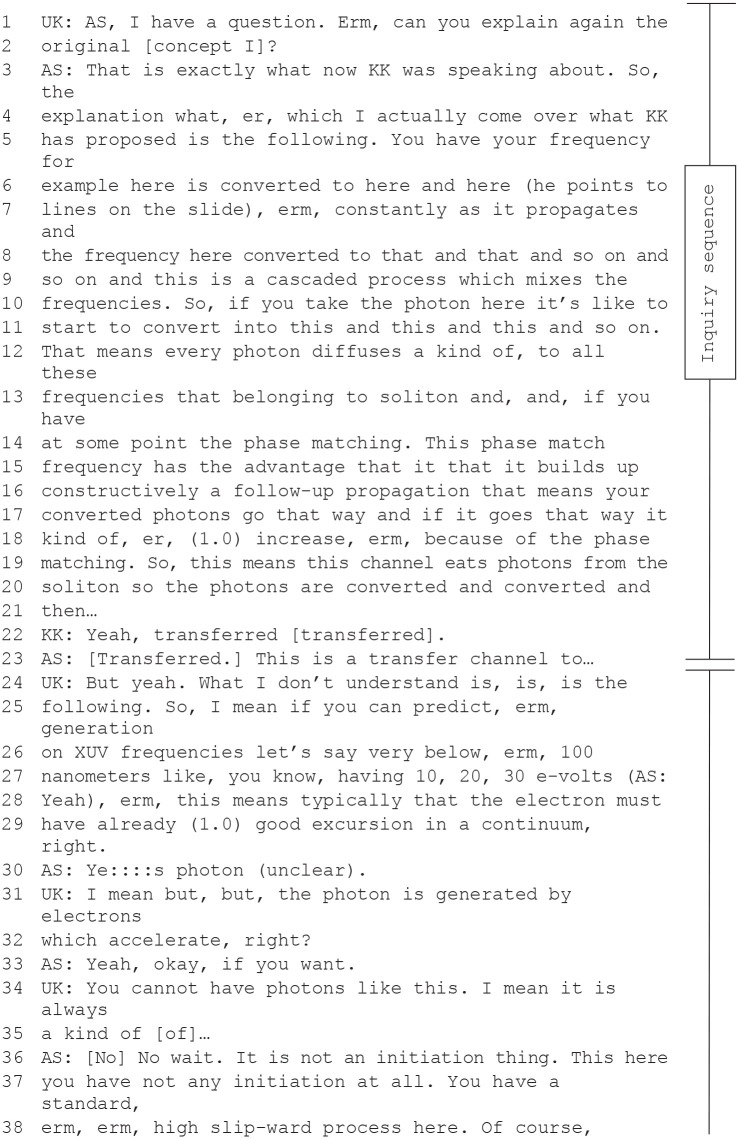



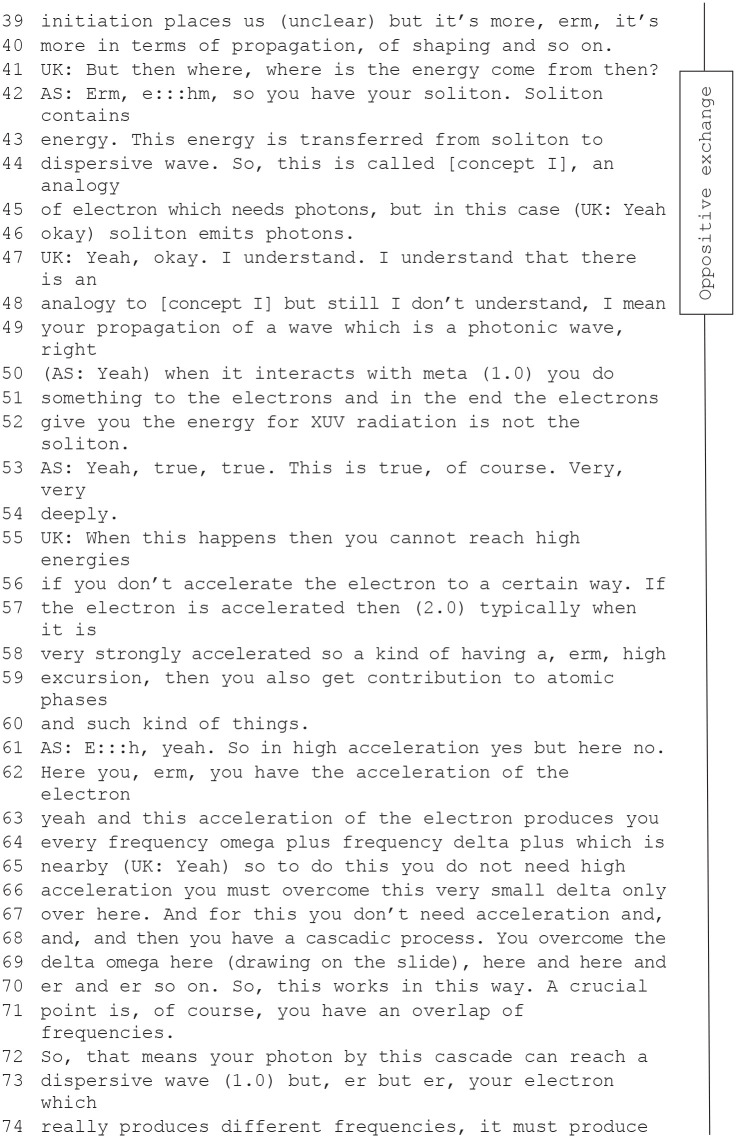



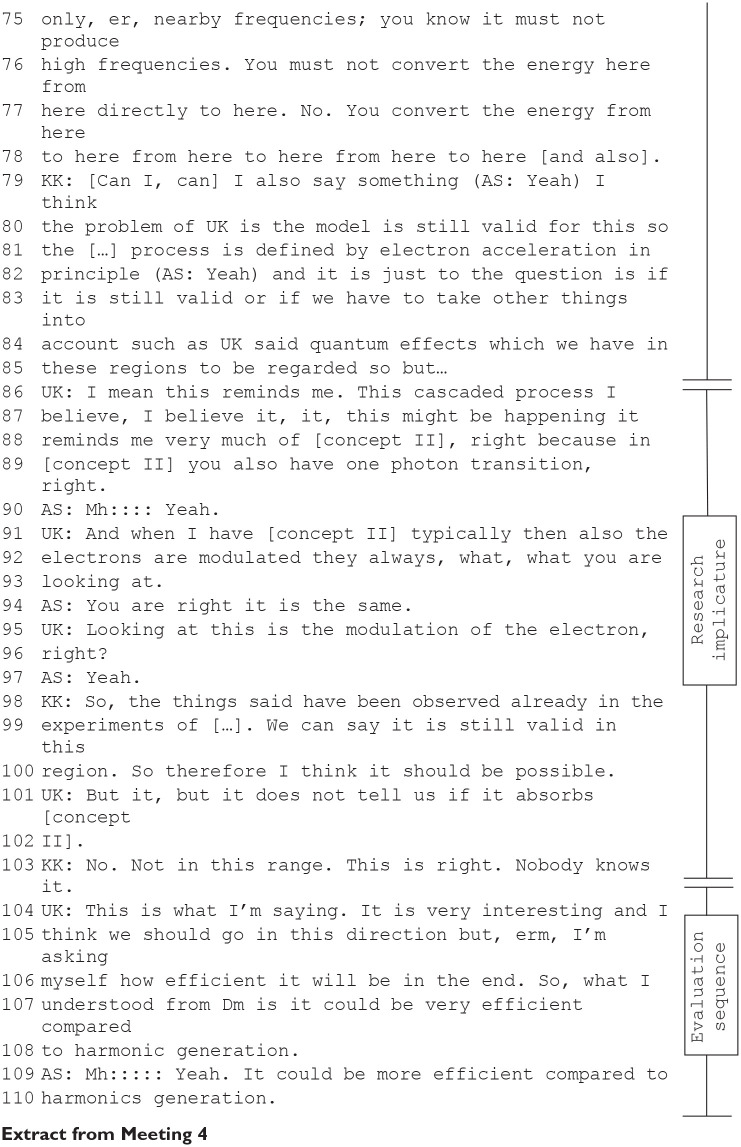


An important feature of the inquiry sequence in the extract of Meeting 39 is that the senior scientist *UK*—who supervises the experimental unit and is not involved in *EJ*’s (doctoral student) research—asks about procedural aspects in order to deliberate on a further research option. He is not primarily interested in how *EJ* generated his results to interpret the data. He instead uncovers in his exchange with *EJ* a research opportunity that goes beyond the presented findings and implications. When the outlines of the new experiment appear in the exchange, the head of the research team (*HD*) intervenes and recommends investigations with a technical infrastructure that is available at the university (in terms of ‘making things work’ in Knorr Cetina, 1981). *HD*’s advice supports *UK*’s research implicature indicating that the research at this point is feasible and not out of reach. This exchange shows how the imaginations shared by individual members in team meetings are kept within the bounds of the team’s research agenda. In regular lab meetings, one could say that research done by one or a few scientists becomes, through reflective talking, an activity of the entire research team.

Such research implicatures also occurred when members of the theoretical unit present new concepts. Presenters usually provided a concept with preliminary findings based on simulations and initial measurements. Such conceptual work gave an idea of what kind of experiments could follow. In the subsequent inquiries and oppositive exchanges, other researchers got acquainted with the idea, reflected upon it, and sometimes produced interactively new insights that implied additional research directions. The discussion in the extract of Meeting 4 illustrates how researchers of the team got hold of the theoretical concept under discussion and connected it to other observations, leading to a research implicature. This part of the discussion starts with an inquiry sequence and then turns into an oppositive exchange.

This discussion involved the three senior scientists *UK, AS*, and *KK*, who produced the most critical inquiries in the observed meetings. In fact, if *AS* was not presenting, he always has questions. However, *UK* and *KK*, in their positions as supervisors of the experimental and the theoretical unit respectively, were active in most exchanges. In this exchange, a research implicature occurs after an extended oppositive exchange between the three scientists. The quote starts when *AS* is clarifying his presented idea against critical inquiries of *UK* and *KK*. But more importantly, this conversation goes beyond the subject of the presentation. In lines 67 to 68, *AS* replaces *UK*’s mentioning of acceleration with the concept of cascades, which he had already used in his presentation to explain why it is not necessary to have an electron with a high frequency. *UK* takes this model of a cascadic process in his turn to bring in a different perspective on the subject. When he shifts the conversation to another concept and its effects on electrons, it becomes apparent that they are now talking about electron absorption in a range nobody knows—a blind spot in the research. Thus, in the oppositive exchange *UK* and *KK* not only got familiar with *AS*’s proposed model, but they also started to think in new directions.

Amann and Knorr Cetina (1989) emphasize that scientists get into oppositive exchange because of their preference for disagreement, which they contrast with agreement talk between physicians and patients. In this respect, oppositive devices function to introduce further readings. In regular lab meetings, we argue that they function as devices to check presented ideas and results. As friendly peers, members of the research team relate these concepts and findings to the current state of research and produce oppositive propositions in order to check the presented line of argument and sometimes to find errors before they are communicated to the scientific community at large. Moreover, in oppositive exchanges participants reflect on the presentation in terms of the research team’s agenda. These interactions not only consolidate the group, but they also inspire research implicatures which might orient the team’s future research.

### Announcements

Finally, the observed meetings were formally closed with the question: ‘Any announcements?’ Often, when there were none, the moderator closed the meeting and all attendees quickly left the online room. In the case of there being announcements, they only took a little time (on average five minutes) compared to the parts of scientific presentations and follow-up discussions (on average 51 minutes). Actually, there were possibly more announcements compared to other years, due to the Covid-19 pandemic. A large portion included information about institutional regulations to deal with the pandemic situation. Other topics were IT security, reference management software, safety regulations in the labs and joint non-scientific events of the research team. While the team did not spend a lot of time on these issues such announcements were also an important device to help build the research team as a whole. Moderators, when asking for announcements, addressed all meeting participants and in doing so outlined again and again who belongs to the research team.

## Discussion and conclusion

In following the regular lab meetings of a research team in optical physics, we found that these are not only used as a bureaucratic form of coordination, control, and rule observation. The team’s leaders used the meetings for announcements, to supervise team members, and to monitor ongoing research, but scheduled gatherings are not restricted to this. Lab meetings are also occasions to report on ongoing experiments, introduce certain topics and discuss findings with a small part of the scientific community—the research team that works on the same topic in different sections, groups and rooms. In these internal meetings, members of the research team come together and get to know what others are doing, and engage in critical exchanges about concepts, experimental set-ups and findings. In the observed meetings, for example, students presented their BA and MA theses, not for oral examinations but as contributions to the team’s research. Moreover, if one compares the oral presentations with the related publications of the presenters, presentations always offered more information on the research, including practical implications based on additional and failed measurements. In short, regular meetings give research teams as a whole the privilege of hearing first-hand about confirmatory results and failures, and discussing how they could be implemented and make conclusions for their own research. Through these meetings undergraduate and graduate students are socialized into research by finding their place in the processes performed by the research team, learning about the team’s research agenda, and becoming acquainted with ways of conducting research in this context (e.g., how to do presentations). In addition, more experienced scientists in the research team gain insights about approaches taken, preliminary findings, mentioned problems and outlooks, which they can deliberate on further and apply in their own research before publication.

Regular lab meetings, of course, are only one of many links between the contingency of research and the linearity of written communication in science. But they are an organizational setting to arrange, check and rewrite proposals and scientific papers additional to the circulation of drafts among critical friends. Various scholars (e.g., Knorr Cetina, 1981; Latour & Woolgar, 1986; Myers, 1989) reported about the common strategy in science to produce a scientific article in an iterative process starting with writing a first version, then asking fellow scientists to review it, and based on their comments, reorganizing and rewriting the draft until it seems publishable. In a further step, journal editors only chose articles that they and reviewers assessed as appropriate and original contributions (Fyfe et al., 2020; Hirschauer, 2010; Myers, 1989). In this production process, we argue that regular meetings of research teams have to be seen as an early or pre-phase. They offer leeway to reflect on the research subject before arguments are written down and presented in a sequential order of sentences and paragraphs. Especially when reading and commenting on a draft, critical friends and reviewers are more concerned with its language tone, writing style and the consistency of arguments than thinking in new directions.

Apart from the mentioned characteristics of regular lab meetings, the main result is that exchanges in these meetings, as a form of shop talk, are critically concerned with presented concepts and findings and include implicatures and recommendations to approve or improve scientific ideas and results. Meetings’ ordered talk does not inhibit collaborative thinking on research issues. Internal meetings rather provide a protected space for the research team as a segment of the scientific community. In this environment, its members prepare presentations in a schematic structure similar to those for scientific conferences, so that others of the team can check their rationale for clarity and mistakes. The others have an opportunity to critically assess the presented concepts and results and control what might be published as an outcome of the team research. In short, on the one hand, regular lab meetings are a pre-step to written communication. On the other hand, disclosing unpublished approaches and findings to the research team in internal meetings is a way of becoming familiar with such concepts and results, to spare others potentially fruitless effort, and, even more, to deliberate on new research. In this form of ‘thinking through talk’ (Amann & Knorr Cetina, 1989) and in particular by research implicatures, researchers take presented ideas and findings a step further and produce hints of signs for supplementary research. For the research team, such talks have the advantage of being ahead of other researchers who have to wait for publication.

Our observations might also be of interest to scholars studying composition of research teams. The findings align with previous studies on the role of experienced researchers in the organization of research teams. Carayol and Matt (2004) and, to some extent, Zhang (2010) showed that research productivity increases with experienced, permanent researchers and decreases with a greater share of inexperienced student researchers. One reason might be that early-career researchers need laboratory training. However, our findings suggest that middle-layer researchers (not full professors) are key to deliberating on research and its outcome. They have the experience and knowledge to take part in oppositive exchanges and to make recommendations to change experimental set-ups as well as to spot research challenges and chances.

Finally, our study also has some limitations. First, the paper concentrated on observations in online meetings. We found similarities between in-person and online meeting situations, but digital media also influences the way participants interact and communicate. Therefore, further research is needed on topical conversation between scientists in online settings compared to face-to-face situations. Second, our assumptions about the importance of regular meetings are somewhat preliminary until other studies provide further findings on meetings in other research contexts. Scholars might investigate how the productivity of research teams differs between those with and without regular lab meetings. Others could take a closer look at the role and function of post-meeting talk in generating research implicatures because they were difficult to observe in online meetings. Moreover, if we take seriously that scheduled meetings of research teams are an environment in which to reflect and to produce research ideas, scholars might examine the relationship between individual scientists’ diverse skills and knowledge and increase scientific productivity or creativity. Innovativeness seems to correlate with organizational settings that are characterized by research team diversity (Carayol & Matt, 2004; Fiore, 2008; Walsh & Lee, 2015). However, how do research teams that are composed of diverse expertise generate creative output? Is it self-organizing? Does it need external impulses? Laboratory studies have shown that scientists who meet in an ad hoc manner in offices, hallways and labs start to exchange details about their ongoing research. Various scholars (e.g., Fleck, 2012; Knorr Cetina, 1981; Kuhn, 1970) have already mentioned that talking about one’s problem in research is an important step to get to new ideas. In addition, we argue that regular internal meetings are a sort of setting that brings scientists together to reflect openly on their own and others’ research: they are a protected space to generate potential research outlooks through talk. Further research has to show whether the observed characteristics of scheduled meetings of research teams vary with disciplines, size and composition. Meetings might function differently if teams are more diverse in their research interests, expertise, and ways of carrying out inquiries.

## Notes

1. Acronyms represent different speakers.
